# Pilot of a blended learning model on medical students’ communication skills in the context of digital innovations

**DOI:** 10.1186/s12909-026-09662-1

**Published:** 2026-06-19

**Authors:** Jonas Töpfer, Sebastian Fritsch, Anja Wollny, Bernd Romeike, Daniela Endlicher, Susanne Schrötter, Sandra Schmitz, Agnieszka Ciężka

**Affiliations:** 1 Institute of General Practice, Rostock University Medical Center, Doberaner Straße 142, 18057 Rostock, Germany; 2 Institute of Anatomy, Rostock University Medical Center, Gertrudenstraße 8, 18057 Rostock, Germany; 3 Dean’s Office for Student Affairs, Medical Education, Rostock University Medical Center, Ernst-Heydemann-Strasse 8, 18057 Rostock, Germany; 4 Dean’s Office for Midwifery Studies, Rostock University Medical Center, Ernst-Heydemann- Str. 8, 18057 Rostock, Germany

**Keywords:** Medical education, Physician–patient communication, Blended learning, Flipped classroom, AI, Gamification

## Abstract

**Background:**

Effective physician–patient communication is essential for high-quality medical care. However, digital transformation and the increasing use of tools based on artificial intelligence (AI) require new competencies that are not yet systematically integrated into medical curricula. Blended learning formats may offer a promising approach to addressing both patient-centered and technology-related communication skills.

**Methods:**

In this pilot study, sixth- and eighth-semester medical students (*n* = 6) participated in an NKLM-based blended learning elective course. The course combined H5P online modules on narrative and AI-related communication with face-to-face sessions involving standardized patients and structured feedback. A mixed-methods evaluation was conducted to assess feasibility and explore learning outcomes. This included a pre–post questionnaire assessing subjective competence gains and an objective structured clinical examination (OSCE) comparing intervention participants with a control group.

**Results:**

The blended learning format was successfully implemented and completed by participating students. Pre–post analyses showed increases across all assessed domains, with larger absolute gains in competencies related to digital innovations than in narrative communication skills. In the OSCE, communication competencies were generally demonstrated across both groups, with the largest performance differences observed in AI-related counselling tasks and consultation closing.

**Conclusions:**

This pilot study supports the feasibility and acceptability of the integrated blended learning course. Exploratory findings suggest potential benefits for both narrative and digital communication competencies, with the strongest gains observed in digital communication competencies. These findings highlight the need to integrate AI- and technology-related communication skills more systematically into medical curricula. Given the exploratory design and small sample size, results should be interpreted as hypothesis-generating.

**Supplementary Information:**

The online version contains supplementary material available at 10.1186/s12909-026-09662-1.

## Background

Successful physician–patient communication is a key determinant of high-quality medical care. Research demonstrates that empathetic and patient-centered communication is positively associated with treatment outcomes [1, 2], coping, self-regulation, treatment adherence and patient engagement, as well as health-related quality of life [2].

Ongoing curricular transformations, accelerated by the COVID-19 pandemic, have highlighted the limitations of purely lecture-based, campus-centered teaching and increased the relevance of digitally-transformed and technology-enhanced learning environments [3–6]. Against this background, research on digital, blended, and hybrid learning approaches in medical education has expanded considerably [7]. Comparative studies suggest that blended learning approaches [8, 9] can be more effective than exclusively online or face-to-face formats and have already been successfully implemented across multiple medical disciplines [10–12]. Reported benefits include higher learner satisfaction, increased engagement, and improved learning outcomes [13–15]. A random controlled trial found that blended learning improved medical students’ communication skills and satisfaction compared with lecture-based teaching [16]. However, there is still limited evidence on how blended learning designs must be structured to optimize learner acceptance, usability, and educational effectiveness [17, 18].

Gamification-based instructional approaches are increasingly used in medical education to enhance learner motivation, engagement, and repeated interaction with learning content [19]. In some cases, they may also support learning outcomes [20–22]. Within this context, interactive digital authoring tools such as H5P (https://h5p.org/) are gaining relevance [23], particularly within flipped classroom designs and formative feedback processes [24]. H5P-based learning activities allow students to engage with content in a self-paced manner, receive immediate feedback, and reflect on their own learning progress. Although empirical findings regarding performance improvements are inconsistent, studies report high user satisfaction, streamlined learning workflows, and didactic benefits [25, 26].

At the same time, standardized patient–based training remains the gold standard for teaching patient-centered communication skills [27]. While conventional lectures and digital tools primarily serve preparation in advance, knowledge acquisition, and reflection, communication competence itself develops through practice in authentic interpersonal interaction [28]. Standardized patient encounters therefore provide an opportunity to apply communication strategies in realistic clinical scenarios, receive structured feedback, and transfer conceptual knowledge into observable communicative behavior. Recent evidence shows, that AI-based tools may further extend this approach by exposing students to virtual patients, digitally mediated communication scenarios, and interactions with increasingly informed, technology-using patients [29].

Taken together, face-to-face, digital, blended and hybrid learning formats each offer distinct educational strengths, but their specific contributions to communication skill acquisition have not yet been systematically aligned with medical communication training. The present blended learning concept was therefore designed to combine these complementary mechanisms: digital modules to support knowledge acquisition and self-reflection, gamified H5P activities to promote engagement and formative learning, AI-related tools to address emerging digital communication competencies, and standardized patient training to enable experiential practice and feedback.

Against this background, the primary objective of this pilot study was to evaluate the feasibility and preliminary educational effects of an NKLM-based blended learning course for medical communication training. The evaluation consisted of a pre–post assessment, an OSCE [30], and qualitative focus groups. However, the present article reports exclusively on the learning design and the results of the quantitative evaluation.

## Methods

### Study design and participants

In the elective course “Medicine Meets AI: Rethinking Medical Communication Training”, sixth- and eighth-semester medical students at the University of Rostock were taught medical communication using a blended learning format. Recruitment for both the elective course and control group was conducted through flyers and university social media channels. In total, eight students initially enrolled; one student withdrew at the beginning of the study for time-related reasons, and another student, although participating in the elective course, was excluded from the pilot analysis due to non-participation in the pre-test and the e-learning components. The sample size was determined by the number of students enrolling in the elective course.

### Self-directed learning phases

The course was designed as a blended learning format combining traditional face-to-face sessions with digital self-directed learning phases. H5P-based learning units (https://h5p.org/) provided students with immediate feedback on the interactive learning content and supported the ongoing assessment of their own learning progress. Content was delivered through interactive input followed by structured reflection and consolidation activities, including quizzes, drag-and-drop tasks, and interactive videos. The digital component comprised three online modules (Table 1):

Module 1 addressed “*Narrative communication*” and introduced key principles of patient-centered physician–patient communication. It covered both theoretical foundations and practical techniques, including active listening, narrative elicitation, and structured consultation opening and closing. Module 2 focused on “*Techniques of structured information gathering*”, introducing the WWSZ technique (Wait-Repeat-Reflect-Summarize) [31] and the four-sides model of communication [32]. Module 3 addressed “*Technological advancements and informed patients*”, covering digital health applications and generative AI (genAI) tools such as chatbots. The modules were delivered via the learning management system ILIAS, providing students with unrestricted access to the sequentially released content.

**Table 1:** Overview of online modules and their alignment with the National Competence-Based Learning Objectives Catalog for Medicine (NKLM) [33]

Module	Chapter	Content	Relation to NKLM* [34]
Module 0: General introduction	0 General introduction	Description of contents, learning objectives, instructions, relevance	/
Module 1: Narrative communication	1.1 Introduction to Module 1	Learning objectives and relevance of the module	/
	1.2 Theoretical concepts	Foundations of narrative communication, its complementary role to evidence-based medicine, distinctions from classic history taking, and principles of shared decision making	VIII.2 − 01 VIII.2-01.1.1 VIII.2-02.6
	1.3 Consultation setting and opening	Structured, empathetic consultation opening and setting, including open-ended questioning and reflection on common introductory pitfalls	VIII.2-02.1.1 VIII.2-02.3
	1.4 Setting the agenda	Structured consultation management, including transparent time management, use of a shared agenda, and its role in patient-centered communication	VIII.2-02.2, VIII.2-02.2.1 VIII.2-02.2.2
	1.5 Listening and understanding	Active listening in physician–patient communication, differentiation from non-active listening and application of related techniques in case-based scenarios	VIII.2-02.1.2 VIII.2-02.1.6 VIII.2-02.2.3 VIII.2-02.2.4
	1.6 Inviting the patient’s story	Narrative elicitation in physician–patient communication, including appropriate timing, independent formulation of questions, and justified selection of topics to be explored	VIII.2-02.1.2 VIII.2-02.2.3 VIII.2-02.2.4
	1.7 Further questioning techniques	Identification and explanation of open and closed questions, evaluation of narrative, open, and funneling techniques as well as active listening in case-based scenarios	VIII.2-02.2.3 VIII.2-02.2.4
	1.8 Ending the conversation	Structured consultation closing, including application of a five-step model, identification of patient-centered closing elements, and recognition of common pitfalls	VIII.2-02.7
Module 2: Techniques of structured information gathering	2.1 Introduction to Module 2	Learning objectives and relevance of the module	/
	2.2 WWSZ technique (Wait-Repeat-Reflect-Summarize)	WWSZ technique, including identification and explanation of its components and structured perception of relevant information in patient encounters	VIII.2-02.1.2 VIII.2-02.1.6 VIII.2-02.2.3 VIII.2-02.2.4
	2.3 Four-sides Model	Four-sides model of communication, including identification of its components, interpretation of physician–patient interactions using the model, and reflection on the impact of one’s own communication	VIII.2-02.1.2 VIII.2-02.2.3
Module 3: Technological advancements and informed patients	3.1 Introduction to Module 3	Learning objectives and relevance of the module	/
	3.2 Generative Artificial Intelligence (genAI)	Application of genAI in medicine, including identification of key use cases and critical reflection on its utilization	VIII.2-06.3 VIII.2-06.3.1 VIII.2-06.3.2
	3.3 “Ada - Your Digital Health Companion”	Understanding the purpose and application of the Ada app in medical contexts	VIII.2-06.3 VIII.2-06.3.1 VIII.2-06.3.2
	3.4 arriba - evidence-based consultation	Purpose, underlying principles, and scope of application of the arriba tool	VIII.2-06.3 VIII.2-06.3.1 VIII.2-06.3.2
	3.5 Character.AI	Design and operational principles of AI-based chatbots	VIII.2-06.3 VIII.2-06.3.1 VIII.2-06.3.2
	3.6 Communicating with informed patients	Respectful and constructive engagement with informed patients, including identification and explanation of communication strategies that foster understanding and trust	VIII.2-02.5.1 VIII.2-02.5.3

*For description of NKLM learning objectives see Appendix 1

### Learning management system analytics

Due to local institutional data protection regulations, individual usage data from ILIAS, such as access frequency, time-on-task, or H5P interaction logs, could not be collected or analyzed. Feasibility and engagement with the online modules were therefore assessed through participant self-report during the course evaluation.

### Face-to-face sessions

**Fig. 1:**
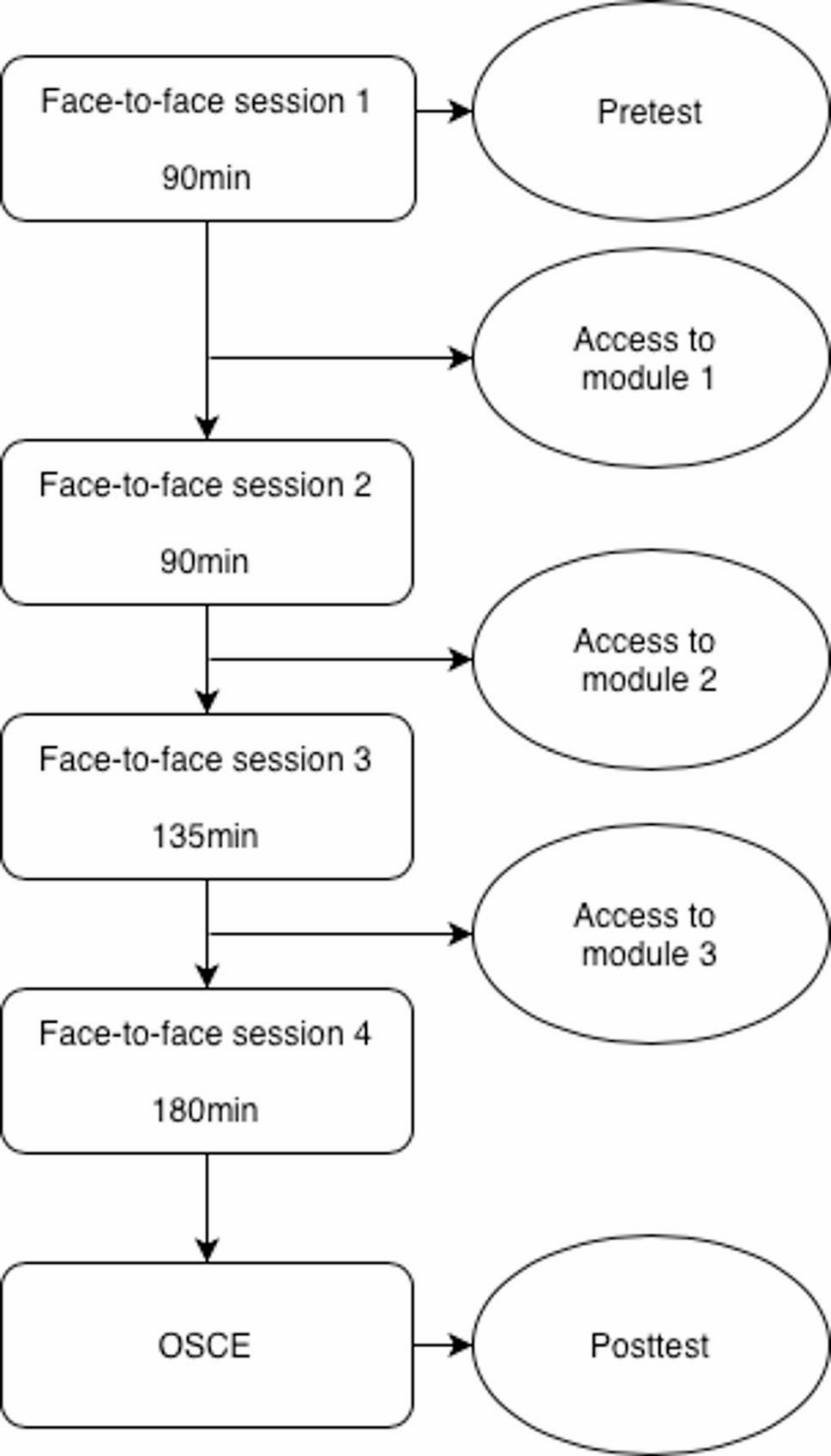
Study flow

The face-to-face component comprised four sessions that were aligned in timing and content with the online modules (Fig. 1). In accordance with the flipped-classroom model, these sessions focused on discussion and consolidation of previously covered content, as well as its application in practice-oriented scenarios.

#### First session (90 min)

The session began with an introduction, clarification of expectations, and presentation of the learning objectives and organizational framework. The pre-test was then administered. Module 1 was subsequently released, initiating the first self-directed learning phase.

#### Second session (90 min, four weeks later)

The session focused on training in narrative communication. Through a series of exercises, students practiced active listening, open-ended questions, targeted questioning and note-taking techniques using common topics (e.g., holiday experiences or illness experiences). Roles alternated between narrator, listener, and observer. The aim of the session was to enhance sensitivity to patient-centered communication and further develop students’ communication competencies. A plenary discussion was conducted to reflect on the experiences.

#### Third session (135 min, two weeks later)

The session focused on communication training with standardized patients. Using uncomplicated cases (cough and low back pain), students conducted general practice history-taking consultations. The focus was on applying the communication techniques introduced in Modules 1 and 2. Facilitators and peers observed the consultations and provided structured feedback.

#### Fourth session (180 min, four weeks later)

The final session focused on practical engagement with digital tools relevant to physician–patient communication. Students explored mobile health applications, including the symptom-assessment app Ada Health [35] and the consultation support tool arriba [36], and applied them in consultation scenarios involving symptom-oriented information gathering, patient education, and communication of individualized risk information. In addition, despite obvious limitations on the use of chatbots in therapeutic communications [37], students interacted with a custom-designed patient chatbot on the Character.AI platform [38] and reflected on symptoms, patient concerns, communication challenges, and the emerging role of AI-mediated communication in healthcare.

### Pre-post assessment

Based on the Communication Skills Attitude Scale (CSAS) [39], general attitudes were assessed. In addition, NKLM-related learning objectives were derived from the online modules. These were used to develop a pre–post questionnaire assessing subjective competence gains in theoretical understanding and applied communication skills, based on Bloom’s taxonomy of educational objectives [40]:

- General attitudes (GA, 9 items): baseline attitudes toward the importance of communication competencies in doctoral practice, openness to technological change in medicine, and self-assessed learning ability [39].
- Theoretical understanding (TU, 35 items**)**: knowledge related to narrative communication, patient-centered communication, questioning techniques, and managing digital innovations.
- Applied communication skills (ACS, 18 items): translation of TU-related into practical communication contexts.

Self-assessments were recorded using a 6-point Likert scale ranging from “strongly disagree” [1] to “strongly agree” [6], assessing agreement with statements (e.g., “I can explain the principles of narrative communication”). Negatively worded items were reverse coded. In addition, sociodemographic data were collected, including gender, age, semester, prior vocational training, and clinical experience.

Items from the three domains were aggregated into a total of 22 mean score scales (Tables 3, 4 and 5). Based on these scale scores, an *absolute gain* was calculated for each scale, reflecting the subjective competence gain from pre-test to post-test. For further analysis, scales were grouped into the categories “*Narrative communication*” and “*Managing digital innovations*,” for which *mean absolute gains* were calculated as the average of the corresponding scale-specific absolute gains.

### OSCE

Practical skills were assessed using an OSCE based on a standardized history-taking scenario involving the case vignette “diarrhea.” Performance of the intervention group was compared with that of a control group of seven medical students from the sixth (*n* = 4) and eighth semester (*n* = 3). Control participants were informed about the general study context but not the case vignette. Trained standardized patients were selected from the professional standardized patient program at the Rostock University Medical Center.

OSCE performance was evaluated using a standardized communication rubric comprising eleven evaluation criteria (Table 2) rated on a 3-point scale (0–2 points). Two teams of two examiners were instructed using a shared assessment framework prior to the examination. Formal inter-rater reliability statistics (e.g., ICC or Cohen’s K) could not be calculated because individual encounters were assessed by separate examiner teams without overlapping ratings of the same performance. However, students were randomly assigned to the study arms, such that potential differences between examiner teams were distributed unsystematically across groups.

**Table 2:** OSCE evaluation criteria

Number	Criterion
1	Friendly Greeting
2	Open Conversation Initiation
3	Agenda Setting
4	Trust-Building Communication
5	Transparent Structure
6	Active Listening
7	Conversational Expansion
8	Content Summary
9	AI Tool Counselling
10	Appropriate Closing
11	Information Gathering

## Results

### Sociodemographic characteristics

A total of six students were included in the analysis (three male, two female, one not reported). Four students were in their sixth semester and two in their eighth semester. The mean age was 24.2 years (SD = 2.99). Two students had completed prior vocational training, and the mean duration of clinical experience was 3.67 (SD = 4.27) weeks.

### Pre-test

Concerning GA, participants reported openness toward the topic of physician–patient communication and interest in learning patient-centered communication techniques. Scale scores for openness to medical change (M = 4.50) and valuation of physician–patient communication (M = 5.63) are presented in Table 3.

Within the domain of TU, students reported confidence levels above four (“somewhat agree”) on the 6-point Likert scale for active listening (M = 4.50) as well as conversation setting, including consultation opening and closing (M = 4.38) (Table 4). In contrast, confidence ratings below this threshold were observed for knowledge of narrative communication, narrative elicitation, questioning techniques and information gathering, agenda setting, and all scales related to managing digital innovations.

In the domain of ACS (Table 5), confidence ratings below four were observed for agenda and conversation structure, information gathering and narrative elicitation, self-reflection in communication, and all scales related to managing digital innovations. Confidence ratings were comparatively higher in conversation setting and initiation (M = 4.33) as well as active listening (M = 4.08).

### Pre-post comparison

Tables 3, 4 and 5 show the absolute gain from pre-test to post-test. With regard to GA (Table 3), only minor differences were observed, which do not indicate clear changes.

**Table 3:** Pre-post comparison of general attitudes

General attitudes (GA)	PRE			POST			Pre-post comparison
	*n*	mean	SD	*n*	mean	SD	d
Valuing of medical communication	6	5.63	0.26	6	5.50	0.32	− 0.13
Self-assessment of communication skills	6	4.08	0.86	6	4.50	0.55	0.42
Openness to changes in medicine	6	4.50	0.72	6	4.67	0.52	0.17

*Abbreviations*: *SD* Standard deviation, *d* (absolute) gain

**Table 4:** Pre-post comparison of theoretical understanding

Theoretical understanding (TU)		PRE			POST			Pre-post comparison
		n	mean	SD	n	mean	SD	d
Narrative communication *MAG =* *1.41*	Knowledge of narrative communication	6	3.00	0.76	6	5.04	0.37	2.04
	Conversation setting (incl. conversation initiation and closing)	6	4.38	0.67	6	5.54	0.29	1.17
	Active listening	6	4.50	0.42	6	5.58	0.44	1.08
	Narrative elicitation	6	3.33	0.84	6	5.06	0.33	1.72
	Questioning techniques and information gathering	6	3.71	0.62	6	4.96	0.33	1.25
	Agenda setting	6	3.72	0.88	6	4.94	0.33	1.22
Managing digital innovations *MAG =* 2.81	genAI and physician-patient communication	6	3.17	1.25	6	5.42	0.49	2.25
	genAI-based apps for communication support	6	2.11	1.07	6	5.50	0.41	3.39
	genAI apps for patient use	6	2.54	1.34	6	5.38	0.34	2.83
	genAI apps for training physician-patient communication	6	2.17	0.98	6	5.06	0.71	2.89
	Technological understanding of genAI	6	2.33	1.37	6	5.00	0.63	2.67

*Abbreviations*: *SD* Standard deviation, *d* (absolute) gain, *MAG* Mean absolute gain, *genAI* Generative artificial intelligence

**Table 5:** Pre-post comparison of applied communication skills

Applied communication skills (ACS)		PRE			POST			Pre-post comparison
		n	mean	SD	n	mean	SD	d
Narrative communication *MAG =* 1,58	Conversation setting and initiation	6	4.33	0.68	6	5.75	0.27	1.42
	Agenda and conversation structure	6	3.33	0.75	6	4.67	0.88	1.33
	Active listening	6	4.08	1.16	6	5.75	0.27	1.67
	Information gathering and narrative elicitation	6	3.04	0.93	6	5.04	0.29	2.00
	Self-reflection in communication	6	3.67	1.37	6	5.17	0.41	1.50
Managing digital innovations *MAG =* 2,55	Technology reflection in medical practice	6	2.83	1.09	6	5.28	0.49	2.44
	Self-directed learning skills in context of technology	6	3.00	1.67	6	5.33	0.52	2.33
	Application of and counselling on digital health applications	6	1.94	0.91	6	4.83	0.46	2.89

*Abbreviations*: *SD* Standard deviation, *d* (absolute) gain, *MAG* Mean absolute gain, *genAI* Generative artificial intelligence

For TU (Table 4), increases were observed across all scales. The MAG (mean absolute gain) of the scales related to narrative communication was 1.41, while the corresponding value for scales related to managing digital innovations was 2.81. The highest absolute gain was identified for genAI-based apps for communication support at 3.39 scale points. The lowest absolute gain was observed for active listening at 1.08 scale points. After the intervention, the minimum scale value was 4.94.

For ACS (Table 5), a similar pattern emerged, with increases across all scales. The MAG for narrative communication skills was 1.58, whereas it was 2.55 for scales related to managing digital innovations. The highest absolute gain was found for digital health applications at 2.89 scale points. The lowest gain, at 1.33 scale points, was observed for agenda and conversation structure.

### OSCE

The OSCE evaluation shows that most basic communication skills (greeting, conversation initiation, conversation setting, active listening, and information gathering) were reliably demonstrated by all participants (Table 6). The highest scores were observed for active listening (criterion 6) and conversation opening (criteria 1 & 2). In contrast, lower scores were found for agenda setting and conversation structure (criteria 3 & 5).

**Table 6:** OSCE evaluation

Criterion	1	2	3	4	5	6	7	8	9	10	11	Total
Intervention	1.8	2.0	0.7	1.8	1.0	2.0	1.5	1.2	1.8	1.5	1.7	17.0
Control	2.0	1.9	0.4	1.7	1.3	1.7	1.3	1.4	0.6	0.7	2.0	15.0
Difference	-0.2	0.1	0.3	0.1	-0.3	0.3	0.2	-0.2	1.2	0.8	-0.3	2.0

The largest differences between the control and intervention group were observed in AI-related competencies and conversation closing (see Table 2 for evaluation criteria). These two areas contributed substantially to the overall difference of two points between the groups.

## Discussion

The primary purpose of this pilot study was to assess the feasibility and acceptability of a novel NKLM-based blended learning course on medical communication training. As a first exploratory evaluation, students’ self-assessments in a pre-post design were combined with a controlled OSCE.

At pre-test, students reported comparatively higher confidence in active listening and consultation initiation and setting [41]. In contrast, lower confidence levels were reported for other narrative communication techniques, including knowledge of narrative communication, agenda setting, narrative elicitation, information gathering, and self-reflection in communication. Although certain communication skills were perceived as challenging prior to training, post-intervention self-assessments indicated confidence ratings near 5 on the 6-point Likert scale. These findings may be interpreted in light of the stage model proposed by Zöll et al. [42], which classifies communication learning objectives according to their complexity and the level of instruction and guidance required to achieve them.

Other studies have likewise identified variation across specific communication domains when using standardized assessment tools such as the SEGUE questionnaire [43, 44]. However, given the small sample size and therefore limited robustness of the findings within individual scales, domain-level comparisons with existing studies should be interpreted with caution. Instead, the present findings primarily highlight the overall positive effect of the blended learning format on the development of narrative communication skills, which is consistent with previous research [16, 45–48].

Notably, competencies related to digital innovations in physician–patient communication showed the lowest baseline confidence levels but the strongest subjective learning gains following the intervention. In particular, students’ theoretical understanding of AI-based communication support tools and confidence levels of the practical use of digital health applications increased in the self-assessment. The OSCE findings support this observation, as the largest performance differences between intervention and control group were observed in AI-related competencies and consultation closing, which largely accounted for the overall group difference.

These findings also highlight the importance of distinguishing between knowledge acquisition, self-perceived competence, and objectively demonstrated communication competence. While theoretical understanding and self-assessed confidence increased across both digital and traditional communication domains, performance-based differences in the OSCE were more limited and most pronounced for AI-related counselling tasks and consultation closing.

Recent research describes the increasing digitalization of healthcare and the integration of AI-based applications as transformative developments that are reshaping the professional roles and competencies expected of physicians [46, 49, 50]. Conversely, students in the present study reported limited confidence in digital aspects of interaction, suggesting a gap between emerging professional demands and current training. This discrepancy highlights the need to integrate digital communication competencies more systematically into medical curricula [13, 46, 51]. In line with this, a growing body of literature advocates the early integration of AI-related competencies within medical education [52–54].

The findings of this pilot study support the feasibility and acceptability of integrating H5P-supported self-directed learning, AI-related content, and face-to-face communication training within a blended learning format. While conclusions regarding effectiveness cannot be drawn from the present exploratory study, the observed learning outcomes provide preliminary signals that warrant further investigation in larger studies. Students first acquired theoretical knowledge through H5P-supported self-directed learning before applying this knowledge in face-to-face seminars. Independent exploration of narrative communication techniques as well as AI-based tools prior to in-class discussion may promote experiential learning and perspective-taking, including adopting the role of a patient using digital communication support systems.

### Limitations

This study is subject to certain methodological limitations. The small sample size highly limits statistical power and generalizability and precluded inferential statistical analyses, resulting in findings that are primarily descriptive, explorative and hypothesis-generating. Also, although OSCE performance was assessed using a standardized communication rubric and examiner training, formal inter-rater reliability could not be established. Furthermore, although the OSCE provided a performance-based measure and was supported by access to an established standardized patient program at Rostock University Medical Center, ensuring a high level of consistency and professionalism in the simulations [55, 56], data collection still relied in part on self-assessments, which may be affected by known biases, particularly discrepancies between perceived and actual competence [57]. In addition, scale construction followed content-based item groupings, and internal consistency was not assessed, limiting the reliability of the measures [58]. Future iterations of the elective course with a larger sample and the use of a standardized questionnaire would therefore be desirable.

In addition, objective learning management system analytics could not be included due to local data protection regulations. Engagement with the digital modules was therefore assessed through participant self-report rather than platform-derived usage metrics. Although all students reported full completion of the online modules and positive experiences with the digital learning activities, objective measures of module usage or interaction intensity were not available.

Moreover, participation in the elective course was voluntary, likely resulting in a self-selection bias toward highly motivated students with positive attitudes toward patient-centered communication. Although the control group was recruited through the same channels, the elective course was additionally advertised via the university course catalogue, which may have contributed to differences in motivation and interest between groups. However, participants were recruited from the same semesters to minimize differences in prior training.

Beyond this selection effect, self-reported competence gains after the intervention may also be influenced by increased awareness and reflection triggered by engagement with communication-related content.

## Conclusion

Overall, the findings of this pilot study should be interpreted as exploratory and hypothesis-generating. Future studies with larger samples are required to further evaluate the sustainable integration of blended learning formats into medical curricula. This study aims to provide a conceptual stimulus for further research on blended learning. It also highlights the potential of integrating digital innovations, such as digital health applications and AI, into medical communication training and may support future curricular development. Complementary qualitative focus groups were conducted within the same project to capture students’ learning experiences and perceptions and will be reported separately. This will allow for a more detailed examination of the course design and its perceived strengths and limitations.

## Supplementary Information

Supplementary Material 1.

Supplementary Material 2.

## Data Availability

The datasets used and/or analyzed during the current study are available from the corresponding author on reasonable request.

## Abbreviations

(gen)AI: (generative) Artificial intelligence
ACS: Applied communication skills
GA: General attitudes
H5P: Tool for creating interactive learning content
ILIAS: Learning management system for online modules
NKLM: National Competence-Based Learning Objectives Catalog for Medicine
OSCE: Objective structured clinical examination
TU: Theoretical understanding
