# Supplementary material for: Pilot of a blended learning model on medical students’ communication skills in the context of digital innovations
Source: BMC Med Educ. 2026 Jun 19;26:1005. doi: 10.1186/s12909-026-09662-1 (PMC13281593; doi:10.1186/s12909-026-09662-1)
Supplement: Supplementary file 1 — Supplementary Material 1. [file 12909_2026_9662_MOESM1_ESM.docx]

**ArPaK: case vignette Diarrhea**

Role name: Mr./Mrs. Schröder

**Information for the Standardized Patient (SP):**

**Current situation**:

You are Mr./Mrs. Schröder. Two days ago, you suddenly developed diarrhea. At the beginning you also vomited twice, but the vomiting has since ceased.
Since yesterday, you have additionally developed headaches and generalized body aches, and you feel increasingly exhausted and weak. You also feel as if you might have a fever, but you have not been able to check your temperature because there is no thermometer in your shared apartment. You suffer from up to ten bouts of watery, light-colored diarrhea every day. This means you have to go to the bathroom two or three times during the night. You have severe abdominal cramps, especially just before diarrhea. You can hardly eat anything and have no appetite. That's why you only consume herbal tea and a few rusks.

At some point, you clicked through an app called Ada on your cell phone, which you knew about from your circle of friends. In the app, you entered various symptoms: **persistent diarrhea, nighttime bowel movements, abdominal pain, loss of appetite, weakness, unintentional weight loss, dark urine, and fever**. After asking several questions, Ada suggested a number of possible causes, including gastrointestinal infection, irritable bowel syndrome, and colon cancer. Since then, you haven't been able to get it out of your mind, especially since your paternal grandfather died young from colon cancer.

As you are slowly becoming worried and feel so weak, you decide to go and see your family doctor.

**Medical history:**

You have not had any significant illnesses to date. You have also never experienced such severe diarrhea before.

**Symptoms:**

You have up to ten watery, light-colored bowel movements per day (two to three of which occur at night). About half an hour before the urge to defecate, you experience severe abdominal cramps around your navel. You then must curl up. You have headaches and aching limbs and feel weak. You have no appetite and have also lost some weight, and your urine is darker than usual. You would prefer to stay in bed all day. However, you currently have a lot to study and are working on your thesis, which requires a great deal of effort and energy that you do not have. You believe you have a fever and cannot stop thinking about cancer.

Urination: less frequent than usual and urine is dark

- **Medication**: none
- **Allergies**: none
- **Hospital stays / surgeries**: none
- **Physician visits**: irregularly for seasonal infections
- **Health-related behavior**: non-smoker, occasional alcohol consumption (beer, wine; approx. 3 times per week), regular exercise at the gym

**Background information to feelings / thoughts / questions:**

- What could it be? Hopefully, nothing serious.
- When will it finally get better?
- I can't afford to be sick right now!
- This is really annoying to me!
- How am I supposed to get my work done?

**Subjective theory of illness:**

- I'm afraid it could be cancer.

**Opener**:

"I've had terrible diarrhea for two days. I can't get out of the bathroom. I feel really sick and exhausted. I think I have a fever too, everything hurts. I've never had this before."

**Other possible interjections during the conversation to bring up the Ada app and the patient's thoughts about cancer:** "I'm really afraid that it's something serious... I tried an app called Ada; do you know it? I had to enter a lot of information, and then it came up with colon cancer, among other things. And my grandfather died of that... I know it's probably an overreaction, but somehow, I can't get it out of my head."

**ArPak: Diarrhea**

Role name: Mr./Mrs. Schröder

**Information for the examiner:**

*15 minutes per student (introduction to the situation + 10 minutes of doctor-patient conversation + brief feedback, if applicable)*

**Further information:**

- No use of examination techniques
- Focus on the application of the following communication models:
  - Narrative conversation techniques
  - Active listening
  - WWSZ

**Task for the Student:** “*Conduct a general medical history interview and apply the communication models you are familiar with. Take a maximum of 10 minutes.”*
