# Supplementary material for: Pilot of a blended learning model on medical students’ communication skills in the context of digital innovations
Source: BMC Med Educ. 2026 Jun 19;26:1005. doi: 10.1186/s12909-026-09662-1 (PMC13281593; doi:10.1186/s12909-026-09662-1)
Supplement: Supplementary file 2 — Supplementary Material 2. [file 12909_2026_9662_MOESM2_ESM.docx]

**APPENDIX**

**Pilot of a blended learning model on medical students’ communication skills in the context of digital innovations**

*Jonas Töpfer**^[[1]](#footnote-1)^, Sebastian Fritsch*1*^[[2]](#footnote-2)^, Anja Wollny*1*, Bernd Romeike**^[[3]](#footnote-3)^, Daniela Endlicher*1*, Susanne Schrötter^[[4]](#footnote-4)^^[[5]](#footnote-5)^, Sandra Schmitz*3*, Agnieszka Ciężka*1

*Corresponding author: Jonas Töpfer, jonastoepfer@web.de, +49 381 494 2492*

**Appendix 1** Description of NKLM learning objectives

| ID | Competency/Learning objective |
| --- | --- |
| VIII.2-01 | Graduates recognize the key role of communication skills in the medical profession and healthcare, and understand that communication is a skill that can be learned. |
| VIII.2-01.1.1 | Explain and reflect on general theoretical principles of communication. |
| VIII.2-02.1.1 | Greet patients, introduce themselves by name and position, and begin with opening questions that create a space for patients to talk. |
| VIII.2-02.1.2 | Adopt a patient-centered (congruent, accepting, and empathetic) attitude, communicate accordingly, and maintain a professional balance between closeness and distance. |
| VIII.2-02.1.6 | Apply techniques for systematic and structured information gathering. |
| VIII.2-02.2 | Structuring the conversation transparently from start to finish. They can ... |
| VIII.2-02.2.1 | Employ time management tailored to different conversation situations and requirements |
| VIII.2-02.2.2 | Set the agenda for the consultation, taking into account the concerns of both the physician and the patient, and communicate this accordingly. |
| VIII.2-02.2.3 | Apply structured conversation techniques and use particular questioning techniques depending on the task at hand. |
| VIII.2-02.2.4 | Recognize and control the focus during the conversation and manage an appropriate balance between physician-centered and patient-centered communication. |
| VIII.2-02.3 | They find an appropriate way to start the conversation and create a conversation situation that is appropriate to the circumstances. |
| VIII.2-02.5.1 | Respectfully assess patients' understanding. |
| VIII.2-02.5.3 | Be guided by the needs and resources of patients with regard to autonomy and responsibility, the amount and completeness of information, and respect their right to refuse information, especially when communicating a diagnosis. |
| VIII.2-02.6 | They shape decision-making processes together with patients or their representatives, taking into account the prerequisites and possible consequences of forming a judgment (participatory decision-making/shared decision making). They can… |
| VIII.2-02.7 | They are able to plan subsequent steps, particularly diagnostic and therapeutic ones, and to conclude a patient consultation. |
| VIII.2-06.3 | They are familiar with and reflect on the use of medical information technologies. They can... |
| VIII.2-06.3.1 | Appropriately incorporate digital media into physician-patient conversations. |
| VIII.2-06.3.2 | Recognize and reflect on the effects of digital applications on physician-patient conversations and the physician-patient relationship. |

1. Institute of General Practice, Rostock University Medical Center, Doberaner Straße 142, 18057 Rostock, Germany. [↑](#footnote-ref-1)
2. Institute of Anatomy, Rostock University Medical Center, Gertrudenstraße 8, 18057 Rostock, Germany. [↑](#footnote-ref-2)
3. Dean’s Office for Student Affairs, Medical Education, Rostock University Medical Center, Ernst-Heydemann-Strasse 8, 18057 Rostock, Germany. [↑](#footnote-ref-3)
4. Institute of Medical Psychology and Medical Sociology, Rostock University Medical Center, Gehlsheimer Str. 20, 18057 Rostock, Germany. [↑](#footnote-ref-4)
5. Dean's Office for Midwifery Studies, Rostock University Medical Center, Ernst-Heydemann-Str. 8, 18057 Rostock, Germany. [↑](#footnote-ref-5)
